# Silk Fibroin-Derived Smart Living Hydrogels for Regenerative Medicine and Organoid Engineering: Bioactive, Adaptive, and Clinically Translatable Platforms

**DOI:** 10.3390/gels11110908

**Published:** 2025-11-13

**Authors:** Asim Mushtaq, Khai Ly Do, Abdul Wahab, Muhammad Yousaf, Abdul Rahman, Hamid Hussain, Muhammad Ali, Pingfan Du, Miao Su

**Affiliations:** 1 College of Textile Science and Engineering (International Silk Institute), Zhejiang Sci-Tech University, Hangzhou 310018, China; asimmushtaq@zstu.edu.cn; 2 Shengzhou Innovation Research Institute, Zhejiang Sci-Tech University, Shaoxing 312400, China; 3 School of Materials Science and Engineering, Zhejiang Sci-Tech University, Hangzhou 310018, China; 4 ZJU-Hangzhou Global Scientific and Technological Innovation Center, Zhejiang University, Hangzhou 311200, China; 5 Optoelectronics Research Centre, School of Science, Minzu University of China, Beijing 100081, China

**Keywords:** silk fibroin, living hydrogel, organoid, regenerative medicine, tissue engineering

## Abstract

Silk fibroin (SF) has evolved from a traditional biopolymer to a leading regenerative medicine material. Its combination of mechanical strength, biocompatibility, tunable degradation, and molecular adaptability makes SF a unique matrix that is both bioactive and intelligent. Advances in hydrogel engineering have transformed SF from a passive scaffold into a smart, living hydrogel. These systems can instruct cell fate, sense microenvironmental signals, and deliver therapeutic signals as needed. By incorporating stem cells, progenitors, or engineered immune and microbial populations, SF hydrogels now serve as synthetic niches for organoid maturation and as adaptive implants for tissue regeneration. These platforms replicate extracellular matrix complexity and evolve with tissue, showing self-healing, shape-memory, and stimuli-responsive properties. Such features are redefining biomaterial–cell interactions. SF hydrogels are used for wound healing, musculoskeletal repair, neural and cardiac patches, and developing scalable organoid models for disease and drug research. Challenges remain in maintaining long-term cell viability, achieving clinical scalability, and meeting regulatory standards. This review explores how advances in SF engineering, synthetic biology, and organoid science are enabling SF-based smart living hydrogels in bridging the gap between research and clinical use.

## 1. Introduction

Hydrogels are now seen as highly versatile biomaterials for tissue reconstruction and regeneration because they hold high water content, have mechanical properties similar to natural tissue, and can be adjusted for different biochemical functions [1]. At first, hydrogels were thought to be passive materials that mainly acted as structural supports for cells, helping them survive and allowing nutrients and waste to move in and out [2,3]. However, recent progress in biomaterials has shown that hydrogels can play an active role in healing [4]. These modified hydrogels can sense changes in their environment, adapt their behavior, and even include living cells or engineered systems that deliver therapeutic signals directly where they are needed. This makes them valuable for regenerative medicine and organoid engineering [5,6].

Silk fibroin (SF) is a natural polymer known for its strength, flexibility, biocompatibility, and ability to break down at a controlled rate, whether it comes from Bombyx mori or is made in the lab [7,8]. Its unique molecular structure, with both crystalline and flexible regions, allows for the tuning of its stiffness, degradation rate, and water retention for different medical uses [9]. SF is also chemically versatile because its amino acid side chains can be modified with nanoparticles, peptides, or growth factors to give it new and responsive properties [8,10]. Furthermore, SF can be made into many forms, such as printable inks [11] scaffolds, or injectable hydrogels [12], which expands its use in regenerative medicine and organoid research [13]. A key advantage of SF-based living hydrogels is their ability to bring together in vivo regenerative medicine and in vitro organoid engineering [14,15]. In living systems, SF scaffolds provide strength, help cells stick, and break down at a controlled rate, which supports tissue integration and repair in bone, cartilage, nerve, and soft tissues [16]. In vitro, they present a defined, reproducible, and non-tumor-derived matrix substitute for systems such as Matrigel, delivering the exact mechanical cues and biochemical gradients required for organoid self-organization, maturation, and disease modeling [17,18]. This bidirectional relevance is uncommon among biomaterials and underscores SF as a common platform that can translate knowledge and methods between laboratory models and the clinic.

In comparison to other natural hydrogels like collagen, gelatin, alginate, or hyaluronic acid, SF offers outstanding strength and versatility [7,19,20]. Its degradation can be tailored from days to months, it is amenable to varied bio-functional modifications, it is optically clear for imaging-based applications, and it already boasts a strong clinical heritage through FDA-approved applications in sutures and meshes [21,22]. Besides it, several studies reported about SF-based composite hydrogels with polymers like polyethylene glycol (PEG), xanthan gum, chitosan, and polydopamine. These combinations enhance the mechanical stability, biocompatibility, and controllable degradation of hydrogels [23,24,25,26]. The application of nanoparticle-loaded SF hydrogels is an interesting research area, which facilitates controlled drug release, enhanced mechanical properties, targeted therapeutic delivery, and tissue engineering [27].

These properties make SF an effective foundational material for developing hybrid smart hydrogels. In these systems, encapsulated cells, engineered microbes, or immune modulators interact with the scaffold to guide tissue morphogenesis and healing [28]. The smart living SF hydrogel represents a bioengineered matrix that integrates extracellular matrix (ECM) structural mimicry, living cellular components, and responsiveness to external stimuli [29,30]. Such hydrogels support wound healing and angiogenesis [31], and also facilitate organoid formation and function, expanding the possibilities for regenerative medicine and precision health [32,33,34]. SF-based living hydrogels stand out in biomaterials science because they are stable, compatible with living tissues, and adaptable. These qualities give them strong potential to improve tissue regeneration in the body and in lab settings.

**Figure 1:**
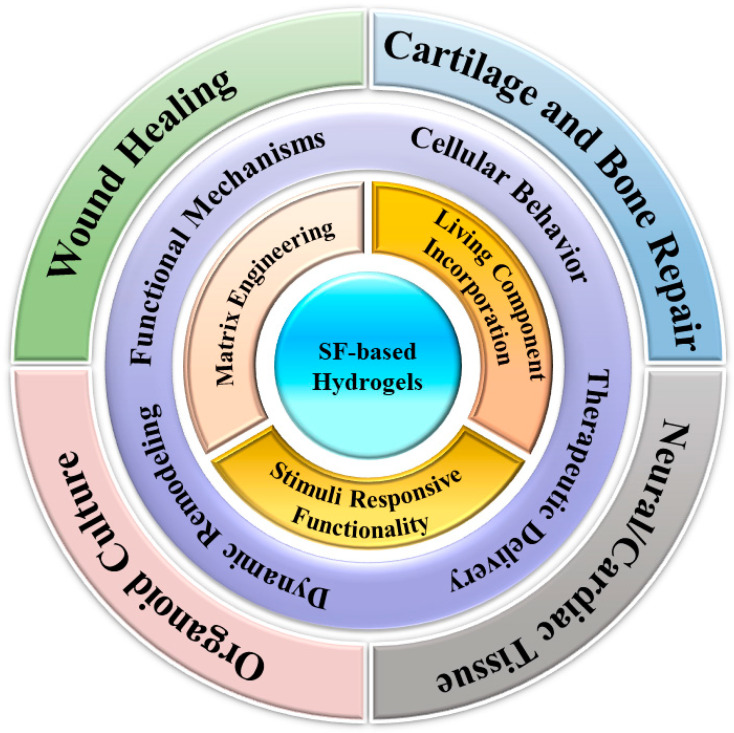
An overview of SF-based hydrogels from functional abilities to biomedical applications.

This review looks at how SF-based smart living hydrogels can be used in organoid engineering and regenerative medicine. It covers how these materials are designed, how they work with living cells, and how they can respond to different signals. The review also explains how these hydrogels affect cell behavior, help deliver treatments, and support tissue repair. Examples include their use in healing wounds, repairing cartilage and bone, engineering neural and heart tissues, and growing organoids (Figure 1). The review discusses challenges like making these materials at scale, ensuring consistent results, and meeting regulatory standards. It also explores future research directions, such as new ways to engineer SF and advances in organoid science. In summary, SF-based living hydrogels have the potential to connect lab research with real-world medical treatments because they are stable, compatible with tissues, and adaptable.

## 2. Design Strategies

SF is a useful and flexible base material for smart living hydrogels because it can be easily adapted for different matrix designs, works well with living biological components, and responds to various stimuli. These qualities make SF both a strong support structure and an active platform that can influence biological processes in complex environments [7]. The next section looks at each of these features and highlights recent research that uses them to create advanced hydrogel systems for tissue regeneration, drug delivery, and organoid modeling.

### 2.1. SF Matrix Engineering

SF has a hierarchical structure made up of ordered crystalline *β*-sheets and less-ordered amorphous regions. This combination gives it a high level of versatility. The crystalline *β*-sheets provide structural stability, mechanical strength, and resistance to enzymatic breakdown. In contrast, the amorphous regions add elasticity, flexibility, and adjustable hydrophilicity [35]. Researchers can precisely engineer the stiffness, degradation rate, porosity, and bioactivity of the resulting hydrogel by adjusting the ratio, distribution, and orientation of these domains through processing methods such as pH adjustment, sonication, enzymatic cross-linking, or physical treatments. Such tuneability makes SF-based matrices extremely versatile for use in a vast array of biomedical applications, from soft, cell-compatible scaffolds for tissue regeneration to robust, long-lasting implants or drug-delivery reservoirs [36].

#### 2.1.1. Polymer Blends for Extracellular Matrix (ECM) Mimicry

The ECM is both a dynamic reservoir and structural framework for signaling that regulates cell adhesion, migration, proliferation, and differentiation. In tissue engineering, it is essential to replicate the complexity of this ECM to maintain real cellular behavior and promote regenerative response. ECM materials, such as Matrigel, present significant limitations, including derivation from tumor sources, batch-to-batch variability, and limited potential for clinical translation. SF, which possesses tunable mechanics, biocompatibility, and processability for mixing, is a robust platform for ECM mimicry. By combining SF with naturally found or synthetic polymers, researchers have been able to create hybrid matrices that imitate biochemical signals, mechanical stability, and dynamic remodeling capability of native ECM, bridging the gap between engineered scaffolds and physiologically relevant microenvironments [30,31].

- Natural polymer blends

*SF–Gelatin Composites*

Combining SF with gelatin produces bio-inks and hydrogels that combine SF’s mechanical strength with gelatin’s cell-adhesive motifs. Photo-crosslinkable SF–gelatin bio-inks have been 3D-printed into structurally stable constructs supporting MSC viability for several weeks and improve osteogenic readouts (e.g., Alkaline phosphatase (ALP) induction and mineral deposition) over gelatin alone. Examples include SF–gelatin bio-inks engineered for high-fidelity printability and preserved cell function, and SF–gelatin hydrogels for skin/bone applications.

**Figure 2:**
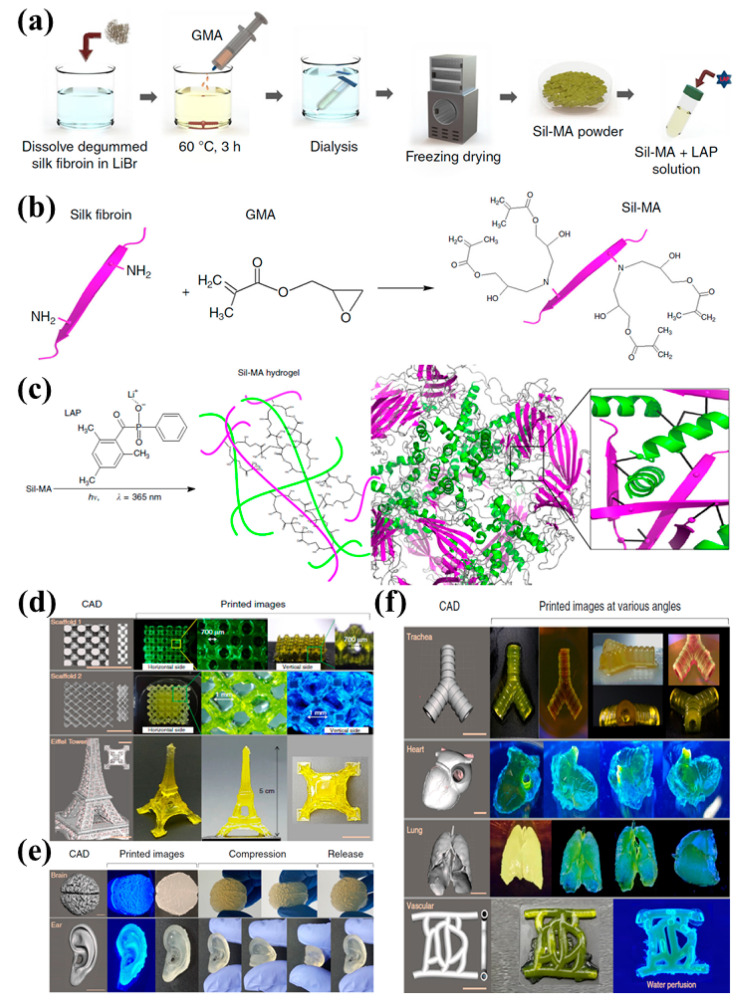
(**a**) Schematic diagram for the SF methacrylation, (**b**) Glycidyl methacrylate (GMA) modification of SF, (**c**) Utilization of lithium phenyl (2,4,6-trimethylbenzoyl) phosphinate (LAP) photo-initiator during polymerization of Sil-MA. (**d**–**f**) Examples of 30% Sil-MA printed with a DLP printer: (**d**) a porous scaffold and a model resembling the Eiffel Tower, (**e**) shapes mimicking an ear and a brain, and (**f**) shapes representing a trachea, heart, lung, and blood vessels. Reproduced with the permission of [11]. Copyright 2018 The Author(s) under the Creative Commons Attribution 4.0 International license (http://creativecommons.org/licenses/by/4.0/ accessed on 10 October 2025).

Schneider et al. demonstrated the potential of composite hydrogels of tyramine-substituted gelatin (G-TA)-cross-linked human placental extracellular matrix (hpcECM) for a sustainable, animal-free bio-ink towards 3D bioprinting of artificial soft tissues [37]. The hybrid system not only preserved the unparalleled bioactivity of placental ECM but also improved biomechanical stability and elasticity, providing a sustainable bio-ink for the 3D bioprinting of artificial soft tissues. In parallel, Chakraborty et al. highlighted the potential of SF as a bio-ink owing to its tunable degradation rates, cytocompatibility, and amenable nature to state-of-the-art 3D and 4D bioprinting strategies [38]. Their review focused on ongoing print fidelity and cytocompatibility concerns, but suggested multicomponent inks as an emerging avenue. Brooks et al. created a photopolymerizable silk and gelatin composite called photo-fibrogel (PFG) by incorporating photoreactive properties with 2-isocyanatoethyl methacrylate (IEM) for tissue engineering and flexible biodevices [39]. This material can be adjusted for swelling, degradation, and electrochemical response, while staying structurally stable. PFG can also be patterned with conductive materials, which makes it useful for bioelectronics and soft robotics. In a similar approach, Asadpour et al. studied scaffolds made from SF, chitosan, and gelatin using freeze-drying [40]. They found that the mix of materials affects porosity, strength, and how quickly the scaffold breaks down. SF gives the structure support, while chitosan and gelatin add water-attracting and biological properties. These combined scaffolds show how mechanical and biological needs can be balanced. For cartilage repair, Li and colleagues developed a macro-porous SF–gelatin hydrogel using enzymatic crosslinking and low-temperature 3D printing [41]. By controlling the size of the pores, the scaffold helps with nutrition and oxygen flow, and guides stem cells to become cartilage, depending on how they are added. Tests in animals showed that this hydrogel can help regenerate cartilage, suggesting it could be useful in orthopedic treatments. In another study, Kim et al. produced SF-methacrylate based bio-ink (Sil-MA) and utilized it to build highly complex organ structures like the brain, ear, heart, trachea, and vessel with remarkable biocompatibility and stability [11]. This makes it promising for tissue and organ engineering as shown in Figure 2. The synthetic route and chemical modifications like glycidyl methacrylate (GMA) and lithium phenyl (2,4,6-trimethylbenzoyl) phosphinate (LAP) to generate Sil-MA hydrogel can be seen in Figure 2a–c. In the next section, it can be observed that 30% Sil-MA has been used to give 3D printed models with the help of a DLP printer (Figure 2d–f).

These investigations highlight a rapidly evolving field where SF acts as a structural and functional template for next-generation bio-inks and scaffolds. Combining SF with gelatin, chitosan, and extracellular matrix derivatives enhances mechanical strength, bioactivity, and tunability, supporting clinical translation. However, challenges remain in balancing degradation rates, ensuring long-term stability, achieving reproducible large-scale production, and developing patient-specific scaffold geometries. Emerging strategies, including photo-patternable composites and eco-friendly ECM-derived inks, are advancing silk-based bio-inks toward broader applications in regenerative medicine and flexible bioelectronics [37,39].

*SF incorporating carboxymethyl chitosan (CMCS) and tannic acid (TA)*

The incorporation of CMCS confers hydrophilicity and intrinsic antibacterial activity, and TA confers phenolic redox/metal-chelating chemistry that confers antibacterial and antioxidant activity and also serves as a mild crosslinker. SF/CMCS hydrogels suppress bacteria (*E. coli*, *S. aureus*) and are pro-healing in vitro and in vivo. TA-reinforced chitosan–silk methacrylate gels also promote re-epithelialization, collagen deposition, and angiogenesis in full-thickness mouse wounds to illustrate how CMCS + TA collaborate with SF in offering infection control and pro-regenerative cues. For example, He et al. prepared tannic acid (TA)-enriched methacrylated chitosan/SF (CSMA/SFMA) hydrogels through a two-step photopolymerization and TA-incubation process [42]. TA acted as a secondary crosslinker with its increasing mechanical properties to five-fold, and as a multifunctional bioactive moiety that provided antioxidant, adhesive, and antimicrobial activity. Hydrogels treated with TA showed enhanced activity against *S. aureus* and *E. coli*, stimulated fibroblast growth, and successfully accelerated wound healing in a mouse full-thickness defect model and are thus promising candidates for advanced wound care. Chen et al. also introduced a low-voltage electrodeposition process (4 V DC) in another study to prepare SF/CMCS composite hydrogels [43]. The SF–CMCS networks with intermolecular hydrogen bonding as the stabilizer formed porous structures as characterized by SEM and rheological analysis. The hydrogels exhibited strong antibacterial activity against *E. coli* and *S. aureus*, enhanced HEK-293 cell proliferation, and stimulation of re-epithelialization and granulation tissue in rat full-thickness wounds, are all instances of their multifunctional use as a cost-effective fabrication method with improved safety.

- Synthetic polymer integration

**Figure 3:**
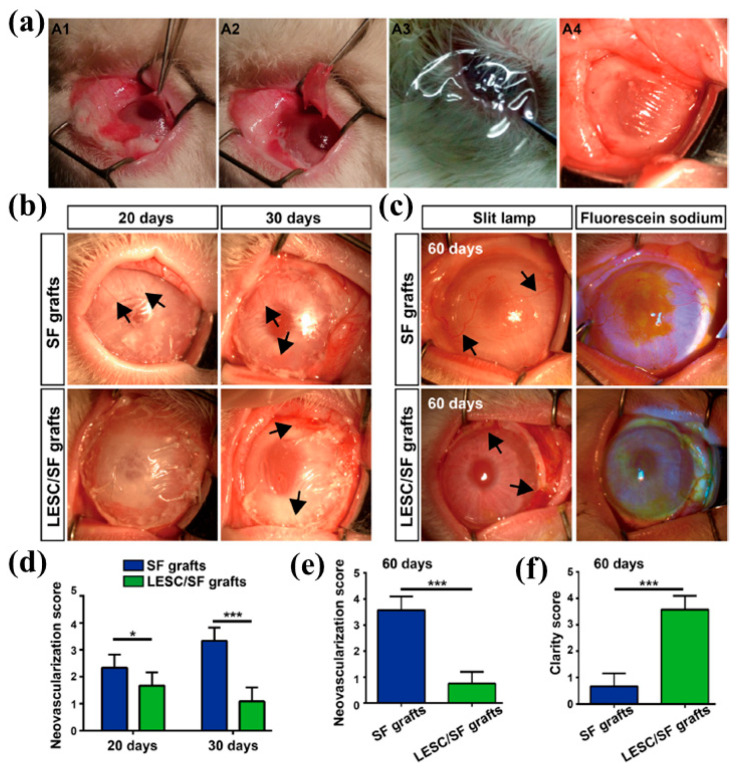
Restoration of limbal stem cell deficiency (LSCD) using LESC/SF graft transplantation. (**a**) The surgical procedure involved removing the corneal vascular pannus (**A1**,**A2**). PEG-modified SF membranes, with or without limbal epithelial stem cells (LESCs), were attached using interrupted 10-0 nylon sutures just beyond the limbus (**A3**,**A4**). (**b**) PEG-modified SF membranes (SF grafts) and LESCs grown on these membranes (LESC/SF grafts) were transplanted onto corneas with LSCD. The images show rabbit corneas at 20 and 30 days after transplantation. (**c**) Images of neovascularization and epithelial defects in rabbit corneas at 60 days after transplantation, with arrows indicating new blood vessels. (**d**–**f**) Neovascularization and clarity scores were measured at 20, 30, and 60 days after transplantation. Data are presented as mean ± SD from three rabbits. Student’s *t* test: * *p* < 0.05; *** *p* < 0.001. Reproduced with the permission of [46]. Copyright 2017 The Author(s) under the Creative Commons Attribution 4.0 International license (http://creativecommons.org/licenses/by/4.0/ accessed on 10 October 2025).

Matrigel is a common but problematic default for organoid culture due to its undefined composition, animal origin, and batch-to-batch variability, which compromise experimental rigor, scale-up, and clinical translation. Considering the deficiencies of Matrigel, chemically defined synthetic scaffolds have emerged as promising alternatives to organoid culture. Polyethylene glycol (PEG)-hydrogels, in particular, offer modular regulation of mechanical stimuli, adhesive ligands, and degradability and have been used to maintain intestinal, lung, and liver organoids with minimalist designs including peptides like RGD, laminin-111, MMP-cleavable crosslinkers, or integrin-binding motifs [44,45]. SF is a high-strength, cyto-compatible biopolymer whose β-sheet content can be adjusted, leading to the mechanical reinforcement and degradable stability. Although SF alone has been sufficient to sustain kidney and cartilage tissue constructs, its PEGylation (PEG-modified SF membranes) has been successfully used in ocular models, wherein PEG-SF membranes served as clear, flexible, and biologically permissive vehicles for limbal epithelial stem cells and restored corneal epithelium in a rabbit model successfully [46], shown in Figure 3. Briefly, Figure 3a(A1–A4) presents the surgical steps from removal of corneal vascular pannus to the addition of PEG-modified SF membranes. After transplantation (20, 30, and 60 days), observations are shown in Figure 3b and Figure 3c, respectively. Furthermore, quantitative measurements are presented in Figure 3d–f.

Although direct applications of PEG-SF matrices in organoid culture are scarce, the synergetic advantages of the chemically defined modularity of PEG and the mechanical and biocompatible properties of SF suggest great promise: PEG-SF composites can potentially offer the mechanics (softness ~200–1300 Pa), protease-induced remodeling, and peptide-mediated adhesion required for organoid morphogenesis, without the complicating complexity of animal-derived hydrogels. In addition, criticisms of human-engineered scaffolds point out that, despite large strides, engineered matrices still fall behind Matrigel in organoid function, at least partly due to weak biodegradability, adhesive ligand complexity, and dynamic spatiotemporal control; thus, the next generation will have to integrate reversible biochemical and biomechanical modulation (e.g., photo-tunable PEG networks, dynamic crosslinkers) to more closely mimic dynamic organoid microenvironments [44].

In brief, polymer blending offers a versatile approach to advance SF from a mechanically intact scaffold to a multi-potential ECM-mimetic matrix. Bio-composites such as SF–gelatin or SF–CMCS–TA introduce hydrogels with bioactive groups that induce osteogenesis, angiogenesis, and antimicrobial shielding, while synthetic composites such as SF–PEG offer designed, controllable platforms highly suitable for organoid culture and translational medicine [47]. Cumulatively, these advances demonstrate that careful polymer blending not only enhances biomimetic fidelity of SF-based hydrogels but also expands their utility to a variety of regenerative and disease-modeling applications.

#### 2.1.2. Bioactive Functionalization

Researchers have greatly improved the bioactivity of SF through targeted chemical modifications aimed at enhancing healing and regenerative processes. Such a novel development is photo-activated SF and collagen-like protein hydrogel (PASCH), a visible-light-crosslinked hybrid hydrogel synthesized by blending SF with a recombinant collagen-like protein (CLP-BS) and riboflavin photo-initiator [48]. In vitro experiments showed that PASCH at a 7:3 SF:CLP ratio formed porous, elastic gels under non-toxic blue light with the advantages of accelerated wound closure, reduced inflammation, excellent biocompatibility, and stability at room temperature as well, and thereby it is an appropriate candidate for diabetic and burn wound treatments and is progressing towards clinical trials. In bone regeneration, SF was effectively functionalized with inorganic nanostructures and growth factors to enhance osteogenesis and angiogenesis. For example, SF–nanohydroxyapatite (nHAp) composites with microspheres encapsulating bone morphogenetic protein-2 (BMP-2) and vascular endothelial growth factor (VEGF) enabled stage-specific, controlled release of growth factors [49]. Together, these findings point to the way SF’s flexibility in composition allows for the creation of extremely multifunctional matrices that can be engineered for sophisticated biological functions as everything from accelerating wound epithelial healing to guiding vascularized bone growth. So, they are just as highly sought after for sophisticated organoid culture systems where instructive, regulatable scaffolds are required.

### 2.2. Incorporation of Living Components

**Figure 4:**
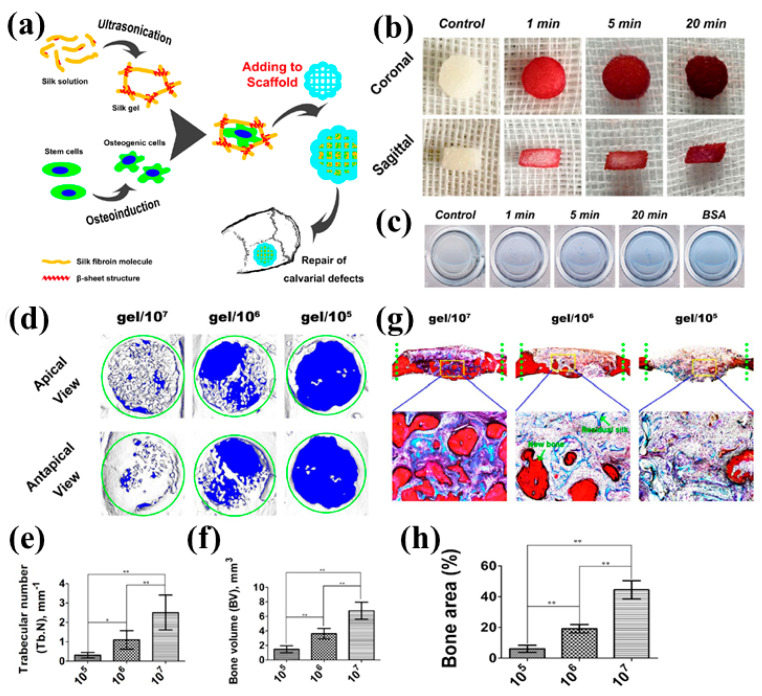
(**a**) Schematic of the fabrication, (**b**) The silk gel and scaffold complex had been placed in alizarin red solution and taken out at various times. The complex was then cut from the coronal and sagittal angles to observe how the solution had permeated. (**c**) The complex was also placed in bovine serum albumin (BSA) solutions for 1, 5, and 20 min, then transferred to distilled water for 24 h to release the BSA (**d**) Representative Micro-CT images show newly formed bone at the defect site from apical and antapical views, with green circles marking the calvarial defect, (**e**) Quantitative analysis of trabecular number, (**f**) Quantitative analysis of new bone volume. * *p* < 0.05; ** *p* < 0.01, (**g**) Van Gieson (VG)-stained histological sections: The green dotted line indicates the calvarial defect, and yellow frames highlight new bone formed by cell-carrying silk gel, (**h**) Quantitative morphometric analysis of new bone area. (** *p* < 0.01). Reproduced with the permission of [50]. Copyright 2017 The Author(s) under the Creative Commons Attribution 4.0 International license (http://creativecommons.org/licenses/by/4.0/ accessed on 10 October 2025).

Incorporation of live cells into SF-based hydrogels advances these biomaterials from inactive scaffolds to dynamic, bioactive microenvironments. Because the gelation processes of SF are so mild and extremely cyto-compatible, mesenchymal stem cell (MSCs) and other progenitor cells can be perfectly incorporated without compromising viability. For instance, silk hydrogels have been reported to preserve the viability of bone marrow-derived mesenchymal stem cell (BMSCs) even when they are embedded with high densities and enhance in vivo osteogenesis in preclinical models of bone defects. It was verified by enhanced alkaline phosphatase (ALP) activity, calcium precipitation, and new bone volume after implantation (Figure 4) [50].

In addition, SF in combination with gelatin promotes MSC proliferation and adhesion: rMSC growth was enabled by SF–gelatin films and osteogenic marker expression (osteopontin), whereas pure SF or gelatin films were less effective [51]. Besides stem-cell embedding, SF matrices are also being explored as well-defined organoid platforms substituted for Matrigel, which exhibit reproducible mechanics and composition and thus higher translational potential. Despite most of the progress having been made with synthetic PEG hydrogels inter-dispersed with organoid cultures, the trend underlines the potential and importance of making SF-based alternatives with controlled properties [45].

The developing concept-based strategies revealed the assimilation of therapeutic living cells, like engineered immune cells or microbes, within the SF hydrogels to generate in situ biological factories. For example, hypothetical encapsulated *E. coli* could be engineered to secrete pro-angiogenic cytokines. These bacteria could be incorporated into SF scaffolds to promote local vascularization and healing, leveraging SF’s mild gelation and structural support. However, direct examples using SF-based systems remain conceptual or in early development. Collectively, these advances highlight the uniqueness of SF for compatibility with living tissue components according to high cell viability, differentiation, nutrient diffusion, and therapeutic action. This places SF-based living hydrogels as a potential powerful platform between bioengineering and regenerative biology.

### 2.3. Smart Responsiveness

One of the defining aspects of next-generation living hydrogels is their ability to actively sense and respond to environmental stimuli, and SF has been found to be an incredibly versatile matrix for the engineering of responsive systems.

*Stimuli-responsive behavior*

SF hydrogels have been widely explored as matrices for on-demand drug delivery with stimulus- and field-responsive release. For example, SF-based scaffolds can be designed to become sensitive to pH alteration, enzyme response, temperature, or applied electric/magnetic fields, and therefore offer strongly regulated drug delivery in a variety of applications from wound healing to cancer therapy [52]. Furthermore, pH- and glucose-sensitive SF composites have also been prepared for diabetes therapy to enable the release of insulin or drugs based on real-time metabolic requirements [53]. Another critical feature is self-healing and structural stability granted by reversible dynamic interactions such as Schiff base linkages, hydrogen bonds, or catechol–metal coordination enabling the hydrogel to heal after mechanical stress, particularly critical for injectable scaffolds for dynamic tissue [54,55].

*Application-driven examples*

In addition to general responsiveness, application-driven developments show how SF hydrogels may be tailored to therapeutic applications. For example, enzymatically Horseradish peroxidase (HRP)/H_2_O_2_ crosslinked SF hydrogels undergo conformational changes that facilitate programmed apoptosis of cancer-like cells in 3D models, providing a dynamic in vitro platform for onco-studies [56,57]. At the same time, researchers are exploring CO_2_-induced gelation as an environmentally friendly and efficient way to create SF hydrogels. This method uses moderate pressure to start the gelation process while maintaining porosity and mechanical stability, which are important for cell infiltration and nutrient diffusion [58].

These developments highlight how SF can serve as the main structure for smart, responsive hydrogel systems. By using reversible crosslinks, sensitivity to metabolic signals, and new gelation methods, SF-based hydrogels can respond to both internal and external changes. This makes them promising for regenerative repair, cancer treatment, and precise control of organoid systems. A general summary of design strategies is given in Table 1.

## 3. Functional Mechanisms of SF-Based Smart Living Hydrogels

SF-based smart living hydrogels are not merely structural matrices but also adaptive bioactive matrices that communicate with living systems. Their functioning mechanisms are defined by the synergy of cell–matrix signaling, drug delivery, and dynamic remodeling. Compared with inert hydrogels, SF-derived living systems provide a dynamic interface to govern cell fate, induce tissue regeneration, and initiate organoid maturation.

### 3.1. Cell–Matrix Signaling

**Figure 5:**
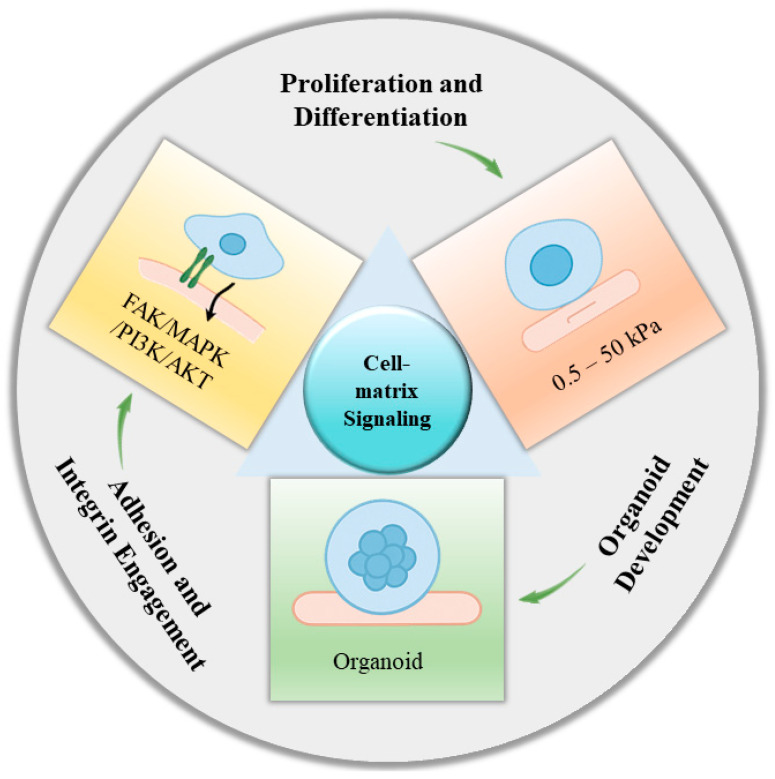
A graphical illustration of cell–matrix signaling.

The way cells interact with the surrounding hydrogel is called cell–matrix signaling. This process is important for controlling how cells stick, grow, move, and develop. Both chemical and physical signals from the hydrogel environment influence cell–matrix signaling. A general overview is given in Figure 5.

*Adhesion and integrin activation*

SF hydrogels interact intensely with cells through integrin-mediated adhesion, which is the basis of cellular perception and response to their immediate surroundings. While SF lacks traditional RGD motifs (Arg–Gly–Asp, peptide sequence) such as in fibronectin, SF does contain RGD-like sequences (for example, Arg–Gly–Glu or Arg–Ala–Asp) and other amino acid domains that are accessible to integrins such as α5β1 and αvβ3. This engagement evokes downstream signal cascades, including focal adhesion kinase (FAK), mitogen-activated protein kinase (MAPK), and phosphoinositide 3-kinase (PI3K)/protein kinase B (AKT) pathways that regulate cell adhesion, cytoskeleton reorganization, survival, and movement [59,60]. For example, in neural tissue engineering, SF hydrogels were found to enhance neural stem cell adhesion and neuronal differentiation compared to inert PEG hydrogels via integrin-mediated signaling that promoted axonal outgrowth and synaptic maturation. This illustrates how SF matrices not only provide physical support but also instructive biochemical cues for effective cell integration [61,62].

*Proliferation and differentiation*

The mechanical stiffness of SF hydrogels can be precisely adjusted within the range of 0.5 to 50 kilopascals (kPa), enabling accurate simulation of heterogeneous-ECM environments [63]. Because matrix mechanics significantly influence stem cell fate, this tunability is essential for directing lineage-specific differentiation. For instance, soft SF matrices (0.5–1 kPa) replicate brain-like elasticity and consequently induce neuronal differentiation by initiating mechanosensitive signaling pathways such as yes-associated protein (YAP)/transcriptional coactivator with PDZ-binding motif (TAZ) suppression. Conversely, stiffer SF matrices (>10 kPa) are more capable of simulating bone tissue biomechanics to support osteogenesis through an increase in cytoskeletal tension and nuclear translocation of osteogenic transcriptional factors like Runx2 [64,65]. Thus, SF hydrogels are not only passive scaffolds but also mechano-transduction regulators, allowing controlled programming of proliferation and differentiation outcomes.

*Organoid development*

Perhaps the most exciting frontier for SF-based hydrogels is in organoid culture systems [66]. Conventional organoid systems are routinely built on tumor-derived and poorly defined ECM extract Matrigel, limiting reproducibility and translational use. SF hydrogels, in contrast, may be bioengineered with laminin-derived bioactive peptides or other ECM-mimetic motifs, creating a defined and tunable microenvironment that supports stem cell self-renewal and differentiation [16]. In a recent work, a DNA–silk fibroin (DNA-SF) hydrogel sustained-release system (DSRGT) was 3D-bioprinted with bone-marrow mesenchymal stem cells to create cartilage organoids (COs), with glucosamine and TD-198946 added to induce matrix formation and chondrogenic differentiation. Unlike the variable mechanical and biochemical cues provided by Matrigel, the SF-based hydrogel enabled fine-tuning of stiffness and functionalization and, consequently, improved maturation of COs. Four-week COs had an ideal phenotype of hyaline cartilage with a high expression of SOX9, type II collagen, and aggrecan, and low expression of fibrocartilage and hypertrophy markers. In vivo, COs induced extensive cartilage restoration in 8 weeks in a rat model of defect via the activation of the MAPK pathway, and transcriptomic profiles were most similar to healthy cartilage. These findings put SF-based hydrogels in the spotlight as a superior candidate to Matrigel for the generation of organoids, with reproducibility, functional tunability, and high potential for translation to cartilage regeneration and beyond [67]. This indicates the promise of SF as a bio-instructive but synthetic alternative for organoid technology, offering translational advantage in disease modeling and regenerative medicine.

### 3.2. In Situ Therapeutic Delivery

SF hydrogels are moving towards active therapeutic platforms from inert scaffolds with dynamic and localized treatment by integrating living cell factories, on-demand release systems, and organoid-based bio-factories.

*Living cell factories*

SF hydrogels ensure a supportive niche for entrapped cells, e.g., adipose MSCs (Ad-MSCs), to continuously secrete therapeutic factors. Silk-fibroin/chitosan hydrogels with pre-loaded Ad-MSCs significantly improved diabetic rat wound healing. The constructs ensured the acceleration of angiogenesis and re-epithelialization with high biomarker expression levels for wound healing, such as VEGF, TGF-β, and EGF, exceeding untreated and hydrogel-alone controls [68,69].

*On-demand therapeutic release*

SF hydrogels may be formulated to respond to pathological stimuli for targeted drug delivery. Green tea catechin (EGCG) grafted on SF hydrogel scaffolds, for instance, imparted in them the capability of scavenging reactive oxygen species (ROS) for accelerated in situ gelation, inhibition of inflammation, and wound healing compared to control hydrogels [70]. One of the examples is methacrylated SF hydrogels containing silver-gallic acid nanoparticles, which controlled macrophage polarization into anti-inflammatory M2 phenotypes and enhanced angiogenesis and antimicrobial activity in diabetic mouse wounds [71].

*Organoid bioreactors*

SF matrices also support long-term culture of progenitor cells into mini-organoids with the capacity to secrete functional biomolecules. HepaRG human hepatocyte progenitors on 3% SF scaffolds in a study had high metabolic activity and facilitated albumin production up to 28 days, indicating that SF is not only compatible with cell viability but also functional maturation with monolayer systems [72].

Together, SF-based hydrogels can administer drugs or biological stimuli actively against disease conditions, release therapeutic factors continuously for regenerative support, and provide mini bioreactors for functional tissue models.

### 3.3. Dynamic Remodeling

SF hydrogels are increasingly being designed to remodel dynamically to accommodate mechanical demand and tissue growth rather than as static implants. Three complementary mechanisms are the foundation of this adaptive behavior: reversible crosslinking, matrix–cell feedback, and stress/shape-memory adaptation [32,73,74,75].

*Reversible crosslinking*

Reversible (dynamic) bonds, whether dynamic covalent chemistries (e.g., Schiff-base, boronate/boronic ester) or highly reversible noncovalent interactions, enable SF networks to self-heal after mechanical damage, relax stresses during matrix deposition, and permit further tissue ingrowth without catastrophic cracking. An example of a good SF-hybrid is the CHA/SF/GCS/DF-PEG hydrogel (coralline hydroxyapatite/SF/glycol chitosan/difunctional PEG) exhibiting rapid self-healing after cutting and prolonged exosome release; the DF-PEG dynamic crosslinks are credited with the fuse-and-recover property of the network and with keeping scaffold integrity under new tissue growth [76]. More broadly, reviews on dynamic-bond hydrogels outline how boronic-ester and other reversible chemistries provide self-healing and adaptive mechanics that are directly applicable to SF hybrid networks [77].

*Matrix–cell feedback*

Dynamic remodeling is also cell-mediated via matrix turnover: cells secrete matrix metalloproteinases (MMPs) and other proteases that locally degrade and soften the hydrogel. Thus, modifying mechanical cues (stiffness, viscoelasticity), cell signaling, and fate decisions leads to a true feedback loop similar to natural wound healing. Several SF studies report on the effects of degradation and mechanical evolution of SF scaffolds on cell proliferation, phenotype, and ECM deposition. For example, research on SF hydrogel degradation illustrates that proteolysis under control alters cell proliferation kinetics and matrix deposition, showing how scaffold degradation and cell activity are coupled [78].

*Shape-memory and stress adaptation*

In mechanically active tissues, shape recovery and stress-adaptive behavior are especially useful. Particularly, SF-based shape-memory organo-hydrogels with semicrystalline micro-inclusions developed organo-hydrogels with shape recovery and high extensibility through semicrystalline micro-inclusions in an SF continuous phase [79]. Such composite strategies (SF + semicrystalline or responsive microdomains) are one way to patch with resistance to cyclic deformation and recovery of architecture and are hence attractive for cardiac or load-bearing applications.

Together, these mechanisms allow SF hydrogels to (i) scaffold extracellular matrix deposition under regeneration without mechanical failure, (ii) deliver evolving mechanical signals that direct cell differentiation and maturation, and (iii) preserve function in mechanically active environments through recoverable mechanics or programmed shape recovery.

## 4. Biomedical & Organoid Applications

### 4.1. Regenerative Medicine

*Wound healing and skin regeneration*

**Figure 6:**
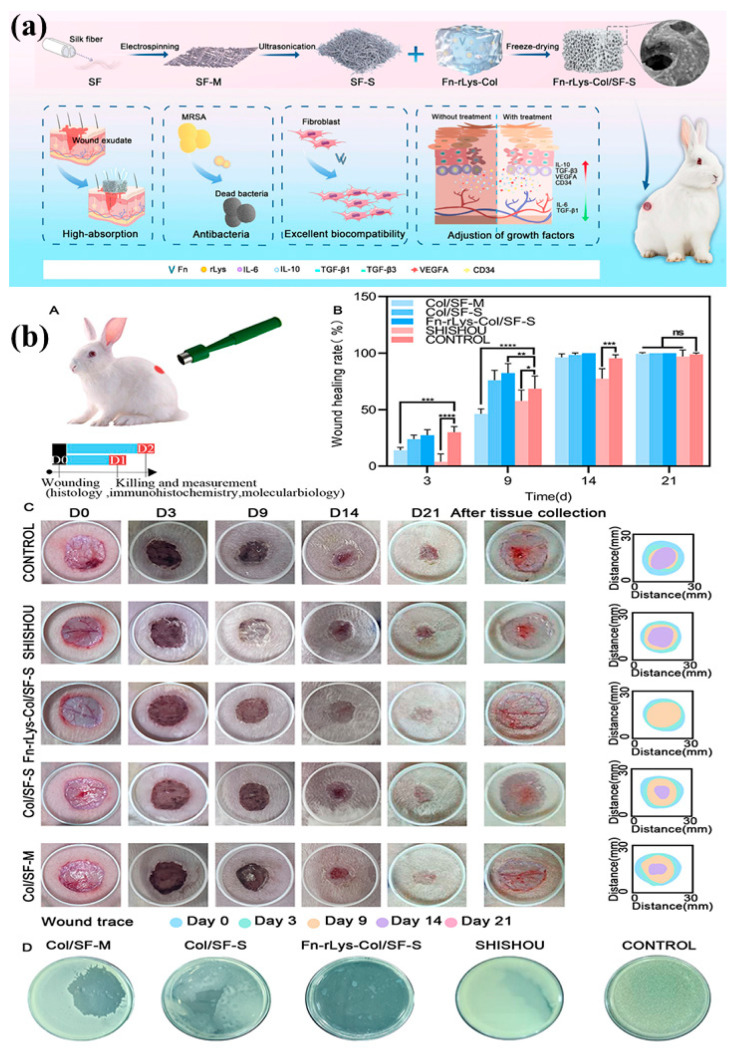
(**a**) An illustration of nano-biomimetic (Fn-rLys-Col/SF-S) for wound healing; (**b**) (**A**) Schematics of New Zealand rabbit’s wound model, (**B**) Rates of wound healing of different groups, (**C**) Overlay images of wounds, (**D**) Bacterial colonies images. Data is displayed as mean ± SD (*n* = 3), * *p* < 0.05, ** *p* < 0.01, *** *p* < 0.001, **** *p* < 0.0001. Reproduced with the permission of [82]. Copyright 2025. Dove Medical Press Ltd., under the license (http://creativecommons.org/licenses/by-nc/4.0/ accessed on 10 October 2025).

Current studies indicate SF-based hydrogels are transforming from passive dressing materials to intelligent, bioactive materials that facilitate active skin regeneration. For example, an epigallocatechin-3-gallate (EGCG)-grafted SF hydrogel, cross-linked through enzymatic methods, exhibited high reactive oxygen species (ROS) scavenging and suppressed inflammation in full-thickness rat skin wounds, accelerating re-epithelialization and angiogenesis compared to unmodified SF dressings [70]. Another innovation is the SF-oxidized salep-carrageenan nanoparticle composite hydrogel with self-healing capability via Schiff-base cross-linking; it was an injectable hydrogel that was stretchy, tissue-adhesive, and promoted bacterial-infected wound healing by its antibacterial and healing-inducing properties [80]. Decellularized SF patches cellularized with human adipose-derived mesenchymal stromal cells have also been shown to improve diabetic mouse wound healing significantly, promoting angiogenesis, ECM remodeling, and re-establishment of skin architecture faster than non-cellularized controls [69]. Additionally, a silk fibroin/fibrin/hyaluronic acid (SF/FIB/HA) scaffold (also known as SFFIBHA) used for critical-size full-thickness burn wounds in rabbits yielded mature epithelium, dermal regeneration, early vascularization, and restoration of skin appendages at 28 days, which was better than a number of traditional dressings for burns [81]. In a study, Liu et al. manufactured nonbiomimetic sponge dressing by fibronectin/lysostaphin-co-loaded silk fibroin modified by collagen (Fn-rLys-Col/SF-S), as shown in Figure 6a. They used the New Zealand rabbit’s wound model (Figure 6b(A)) and the outcomes revealed the promising efficiency of wound healing via ECM mimicking (Figure 6b(B,C)). Furthermore, antibacterial results depicted the efficacy of Fn-rLys-Col/SF-S, as presented in Figure 6b(D).

These studies show that SF hydrogels, especially when combined with live or bioactive ingredients such as stem cells, ROS scavengers, or antibacterial agents, play an active role in healing. They support sustained delivery of healing factors, help cells survive, promote ECM remodeling, and reduce inflammation. As a result, they often perform better than most synthetic or inert dressings in both healing outcomes and their potential for real-world use.

*Bone and Cartilage Regeneration*

SF hydrogels show strong promise for osteochondral regeneration because their adjustable viscoelasticity and ability to remodel more closely resemble the natural ECM than static synthetic gels. In bone tissue regeneration, injectable SF–inorganic hybrid hydrogels like regenerated SF/laponite (RSF/LAP) improved cell adhesion and promoted bone growth and blood vessel formation in a rat calvarial defect model. Adding LAP activated AKT signaling and led to better bone induction than pure SF [83]. Another study developed a porous SF microcarrier with laponite, which released magnesium ions (Mg^2+^) steadily to support bone formation [84]. The optimal structure had about 76% porosity and 25 μm pore size, which could be adjusted by changing the freezing temperature and LAP content. The SF-LAP microcarrier maintained a stable release of Mg^2+^ (1.2–2.3 mM) and a slightly alkaline environment over six weeks of in vitro degradation, both of which supported bone regeneration. Cells grown in these carriers showed higher alkaline phosphatase activity and increased expression of bone-related markers compared to those on plain SF scaffolds. The findings show that SF-LAP microcarriers provide a biodegradable platform that supports bone growth and can be used to repair bone defects and deliver cells.

**Figure 7:**
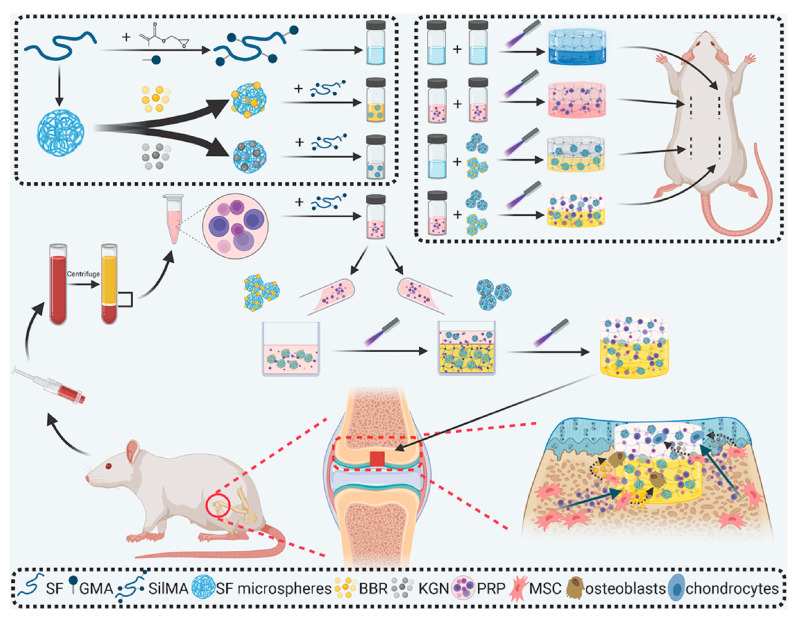
An overview of modified bilayer SilMA in osteochondral repair. Reproduced with the permission of [89]. Copyright 2022 The Authors. Published by Elsevier Ltd. Under the CC BY-NC-ND license (http://creativecommons.org/licenses/by-nc-nd/4.0/ accessed on 10 October 2025).

In a similar way, photo-crosslinkable SF derivatives like silk methacrylate (Sil-MA) or SF-GelMA composites allow for minimally invasive delivery and precise repair of defects. They also keep high printability and can encapsulate cells well for bone formation [85,86,87]. A brief schematic overview of Sil-MA hydrogel from synthesis to bone regeneration is given in Figure 7. The study of Wang et al. revealed the controlled dual delivery of BMP-2 and VEGF by using the scaffold of SF-hydroxyapatite. Briefly, VEGF was released rapidly for initial angiogenesis induction, and BMP-2 was released slowly for osteogenic differentiation and successfully achieved complete bone bridging in rat calvarial defect models within 12 weeks [49]. Similarly, in another study, sonication-treated injectable SF hydrogels loaded with VEGF_165_ and BMP-2 were utilized in a rabbit maxillary sinus floor augmentation model; the two-factor hydrogels caused increased tissue penetration, vascularization, and formation of new bone in situ, with the VEGF–BMP-2 combination being better than gels that released each factor alone [88].

In cartilage tissue engineering, SF hydrogels enable a porous and hydrated microenvironment that preserves chondrocyte viability, glycosaminoglycan (GAG) deposition, and hyaline-like cartilage production. A recent review found that SF-hybrid and SF-bioactive small molecule formulations increase SRY (sex-determining region Y)-box 9 (SOX9) and type II collagen expression while reducing hypertrophic markers. This suggests that SF can help guide cells toward mature cartilage types [90]. Dynamic SF composites with reversible covalent bonds or polyethylene glycol (PEG)-based linkers also show self-healing abilities. These qualities help hydrogels withstand repeated compression without breaking down and support ECM deposition, both of which are important for cartilage that experiences regular mechanical stress [91]. Collectively, these developments indicate that smart living hydrogels based on SF outperform static scaffolds by supporting osteogenesis with vascularization and stable cartilage reconstruction, thereby providing an adaptive biomaterial platform for osteochondral repair.

*Neural Regeneration*

**Figure 8:**
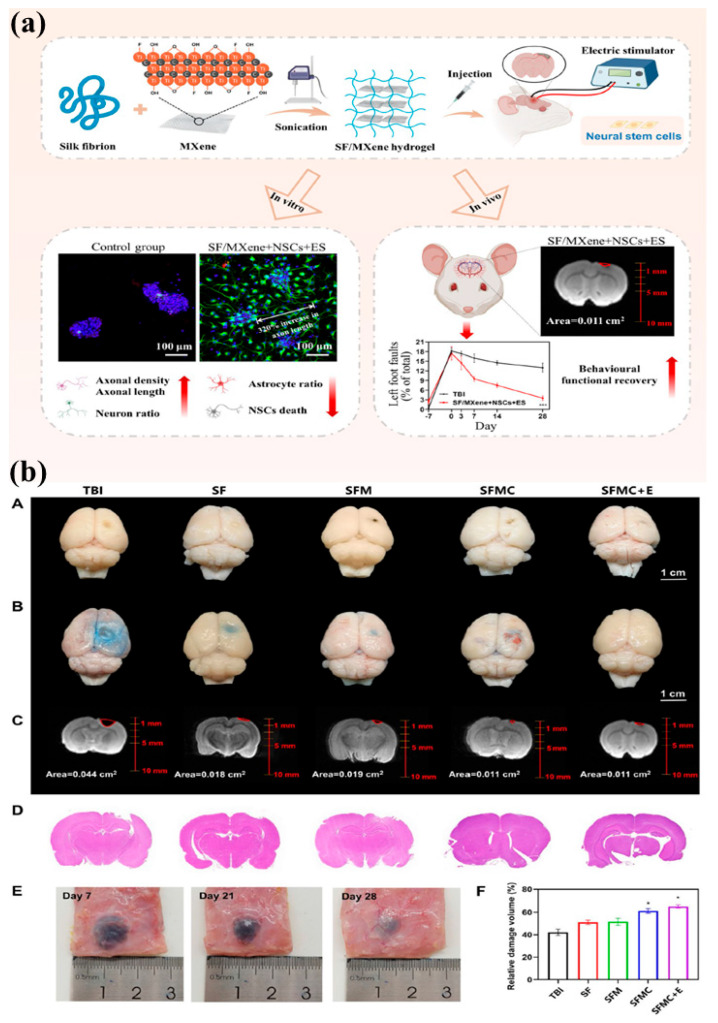
(**a**) Graphical representation of SF/MXene hydrogel for neural regeneration, (**b**) Evans blue staining, NMR imaging, and Hematoxylin and Eosin (H&E) staining were used to assess lesion areas after different treatments. (**A**) Digital images show the groups after TBI treatment. (**B**) Evans blue extravasation in the right brain is shown for each group. (**C**) NMR images display lesion areas in TBI rat brains. (**D**) H&E staining highlights the lesion areas. (**E**) Subcutaneous hydrogels are shown on days 7, 21, and 28. (**F)** Lesion volume is quantitatively analyzed. (±SD, *n* = 3; * *p* < 0.05). Reproduced with the permission of [95]. Copyright 2024 The Authors, under the Creative Commons Attribution 4.0 International license (http://creativecommons.org/licenses/by/4.0/ accessed on 10 October 2025).

Neural regeneration depends on creating an environment that closely resembles the natural nervous system, providing the right bioelectrical and biochemical signals [92,93]. SF-based hydrogels have proven to be promising biomaterials for this, primarily because of their biocompatibility and ability to support the critical aspects of neural progenitor cell (NPC) functionality, including adhesion, proliferation, and differentiation [94]. Recent efforts focus on enhancing these functionalities by integrating conductive fillers such as MXene and polypyrrole to restore essential electrophysiological cues [95,96]. This approach addresses major challenges in neural regeneration, especially the central nervous system’s limited ability to recover after injury or disease [97,98]. Hydrogels made from Bombyx mori SF provide a strong, biocompatible scaffold that acts as a temporary extracellular matrix for neural progenitor cells [94]. The ECM gives both structure and signals that help guide how cells develop [99]. In lab studies, SF hydrogels support the growth, specialization, and survival of neural progenitor cells, particularly when coated with laminin to enhance their biological effects [94]. These hydrogels can also be adjusted to match the stiffness of neural tissue, which is important for how cells behave. The process of neural progenitor cell differentiation depends on several signaling pathways and factors [93,100]. For example, vascular endothelial growth factor and low-density lipoprotein receptor-related protein trigger pathways like Ras-Raf-MEK-ERK and PI3K-AKT, which are key for cell proliferation and differentiation [93]. Wnt/*β*-catenin signaling also helps by encouraging neural progenitor cells to multiply and mature [100,101]. Growth factors such as brain-derived neurotrophic factor and neurotrophin-3 help neurons survive, develop, and expand, and can be added to collagen-based materials to improve neural repair [99]. Electrical signals are essential for how the nervous system works and heals. To mimic this environment, conductive biomaterials are needed [93,102]. Adding materials like MXene and polypyrrole to SF hydrogels helps meet this need [95,96,103]. MXenes are a group of two-dimensional materials with good electrical conductivity and biocompatibility [95]. Polypyrrole is another commonly used conductive polymer that, when added to hydrogels, greatly improves their ability to conduct electricity [103,104]. For example, adding polypyrrole to alginate hydrogels can enhance neural differentiation by providing both electrical conductivity and antioxidant benefits [103]. Such conductive hydrogels facilitate electrical stimulation, which has been employed to direct neural stem cell differentiation along neuronal lineages and amplify neurogenesis [105,106]. The inclusion of these electroconductive materials into hydrogels facilitates the development of platforms that are able to supply electrical stimulation directly to the neural cells, controlling their differentiation and functional integration [95,104]. It has been demonstrated that electroconductive hyaluronic acid hydrogels incorporating carbon nanotube and polypyrrole can enhance neurogenesis of human neural stem cells [104]. SF/MXene conductive hydrogels with injectable properties have also been fabricated with the sole purpose of electrically stimulating neural stem cells for neuronal differentiation, holding promise for the treatment of brain damage [95] as presented in Figure 8a. In detail, Figure 8b(A–C) depict the digital images, Evan blue images and NMR images, of groups after treatment, respectively. These images clearly show the lesion areas in the brains of TBI rats. Similarly lesion areas can also be seen in H&E staining (Figure 8b(D)). Finally, subcutaneous hydrogels at 7, 21, and 28 days and the quantitative volume of lesions are presented in Figure 8b(E) and Figure 8b(F), respectively.

Additionally, neural regeneration microenvironment involves intricate cellular crosstalk. Astrocytes, microglia, and oligodendrocytes all play significant roles [107,108]. Astrocytes nourish and support neurons, and oligodendrocytes myelinate axons, which is required for fast transmission of nerve impulses [107,109]. Demyelination can lead to neurological disease, and remyelination is thus a key component of neural regeneration [92,109]. Oligodendrocytes are formed when neural progenitor cells differentiate, which is regulated by neurotrophins and netrins, and by microglia and Schwann cell cues [93]. Biomaterials can be designed to regulate oligodendrocyte differentiation, and there is therapeutic potential for spinal cord injury (SCI) and other disorders [110].

Synthesis of the next-generation biomaterial scaffolds, like multi-functional artificial niches, aims to overcome stem cell recruitment limitation, senescence delay, and stimulation of neural differentiation [111,112]. These artificially synthesized scaffolds are able to mimic native tissue morphology and physiology and provide a platform for optimal cell-material interaction [113]. For instance, a multi-functional artificial niche can encapsulate drug delivery function (e.g., CXCL12 (C-X-C motif chemokine ligand 12) for recruitment, salvianolic acid A for anti-senescence) and physical signals from multi-channel matrices to promote neural differentiation and accelerated nerve regeneration [111]. New fabrication methods such as 3D bioprinting now make it possible to create advanced collagen-based structures with precise shapes and compositions for neural tissue engineering [92,99]. The nervous system relies on a careful balance of signaling pathways to guide the development and maintenance of progenitor cells [114,115]. For instance, Notch signaling helps maintain progenitor cell self-renewal, while its suppression leads to cell differentiation [116]. Bone morphogenetic proteins (BMPs) and Wnt signaling are also key in regulating the balance between differentiation and growth of neuroepithelial cells [117]. By studying and adjusting these pathways with specially designed SF-based hydrogels that include conductive materials, researchers are making important progress toward effective neural regeneration therapies. Ultimately, the aim is to develop systems that support functional recovery by providing both the necessary structural and biochemical support, as well as essential electrical stimulation [92,93].

*Cardiac and Vascular Tissue Repair*

**Figure 9:**
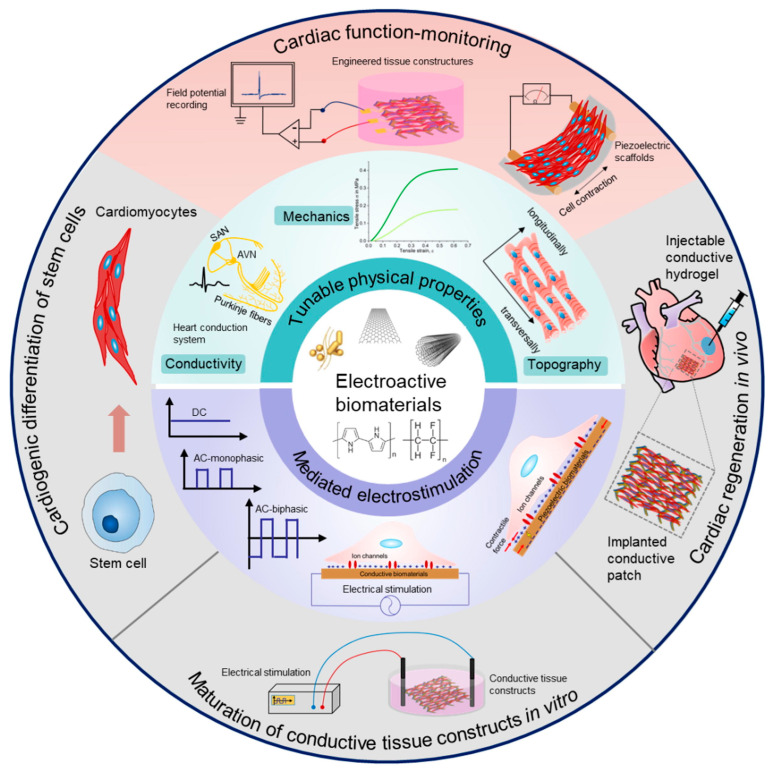
A graphical presentation of electroactive biomaterials for cardiac regeneration and conductive tissues repair. Reproduced with the permission of [118]. Copyright 2023 The Authors. under the CC BY license (http://creativecommons.org/licenses/by/4.0/ accessed on 10 October 2025).

Regenerating vascular and cardiac tissues is especially challenging, especially after events like heart attacks or damage to blood vessels. Recently, SF-based hydrogels have gained attention as promising materials in this area because they combine strength, easy injection, and support for cell growth and integration, along with electrical activity [118,119,120]. These hydrogels aim to improve the complex environment of heart and blood vessel tissues, which need precise electrical and chemical signals to work and heal properly. After a heart attack, heart muscle cells are lost for good, leading to scar tissue and reduced heart function. Standard treatments often cannot fully restore the heart’s original tissue or its ability to contract. Electroactive materials, such as SF hydrogels with conductive additives, help provide the electrical signals needed for heart repair and monitoring. Their physical properties, like conductivity, elasticity, and surface structure, can be adjusted to influence how cells behave and how tissues function [118]. For heart tissues repair, injectable conductive hydrogels are especially promising because they can be delivered right into the damaged area to help start the healing process [118,120]. Hydrogels can be made into implanted conductive patches that help support both the mechanical and electrical aspects of tissue repair. Mediating electrostimulation is important because cardiomyocytes respond to electrical signals, which can help stem cells develop into mature heart cells and improve the growth of conductive tissue in the lab. For instance, electroactive biomaterials can encourage stem cells to become heart cells by providing the right conductivity and electrical signals. These materials are also useful for monitoring heart function. Engineered tissues with these biomaterials can record the electrical activity of heart tissue models. Piezoelectric scaffolds, which convert mechanical energy into electrical signals and the other way around, allow for monitoring heart function without invasive procedures [118]. A graphical illustration is given in Figure 9. Such a function allows for continuous monitoring of the healing process and functional integration of new tissues. For vascular tissue repair, SF-based hydrogels can potentially provide a structural framework that enables the repair of vascular defects. While materials discussed here are directed primarily towards cardiac and neural regeneration, ideas of mechanical resilience and cell delivery are highly transferable. For instance, a conductive hydrogel-coated decellularized umbilical cord has been proposed as a nerve conduit, and it possesses excellent mechanical properties, conductivity, and biocompatibility suitable for peripheral nerve regeneration [121].

This illustrates how conductive hydrogels can provide a nurturing, electrically responsive environment for cell growth and regeneration in other excitable tissues, as is the case with vascular repair. The mechanical properties of SF hydrogels, specifically elasticity and stiffness, can be tailored to replicate the mechanical requirements of blood vessels and provide structural support during healing [118]. Additionally, the capacity of these hydrogels to deliver cells, such as endothelial or vascular smooth muscle cells, is essential for re-endothelialization and restoration of vascular function. Injectable hydrogels, including 3,4-dihydroxyphenylalanine (DOPA)-grafted chitosan and designer peptides, demonstrate enhanced adhesiveness and self-healing properties, which facilitate integration with host tissues and maintain mechanical stability under dynamic physiological conditions such as those present in the heart or blood vessels [119]. One example is an injectable, self-healing, electroconductive ECM-based hydrogel developed to help repair tissue after spinal cord injuries. This hydrogel is biocompatible and supports both cell differentiation and axon growth, which makes it useful for cardiac and vascular treatments [120]. Its self-healing ability is especially helpful in areas that experience ongoing mechanical stress [119,120]. Adding features like biodegradable piezoelectric nanogenerators (PENGs), which allow ultrasound-activated electrical stimulation inside the body, could further support cardiac and vascular repair by helping and tracking tissue regeneration without wires. These systems can provide ongoing electrical stimulation to encourage healing in a less invasive way, addressing major problems with current long-term electrical stimulation methods, such as infection risk and the need for additional procedures [122].

Creating advanced SF-based hydrogels that are strong, deliver cells efficiently, and electroconductivity is an important step forward for repairing heart and blood vessel tissues. By mimicking the body’s natural environment, these materials aim to help tissue grow back, restore normal function, and lead to better results for patients in complex tissue engineering situations.

### 4.2. Organoid Culture Platforms

SF hydrogels are being used more often as scaffolds in organoid culture because they can create artificial basement membranes that closely resemble the natural ECM. Unlike animal-based matrices such as Matrigel, which can vary from batch to batch and have an unclear composition, SF hydrogels give researchers precise control over their mechanical and biochemical features [123,124]. This means scientists can set specific levels of stiffness and viscoelasticity, which are key for directing how cells organize and polarize. This makes organoid models more similar to real tissues [124]. By adding certain ECM molecules to SF, researchers can guide how cells differentiate and arrange themselves, resulting in organoids that better match the structure and function of native tissues. SF hydrogels are also clear, so researchers can watch and image organoid development as it happens, which helps in studying complex biological processes and cell interactions over time [125]. This precision in engineering holds the promise of greater reproducibility of organoid research, redressing one of the most important limitations of previous culture systems.

**Figure 10:**
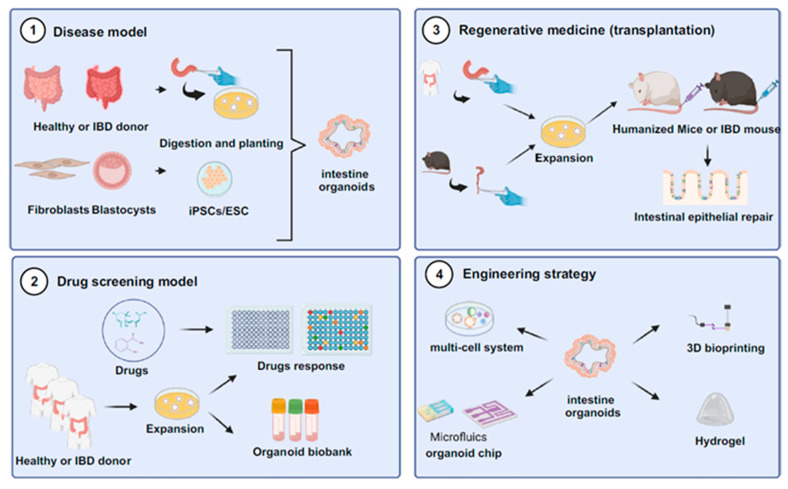
An overview of applications of intestinal organoids. Reproduced with the permission of [126]. Copyright 2024 The Authors, under the Creative Commons Attribution 4.0 International license (http://creativecommons.org/licenses/by/4.0/ accessed on 10 October 2025).

One of the biggest hurdles to generating functional organoids, especially for large or complicated tissues, is the formation of a perfusable vascular bed for sufficient nutrient and oxygen delivery and maximum waste removal. SF hydrogels fill this gap by actively inducing vascularization and allowing long-term maturation of organoids. Experiments have demonstrated that co-culture of endothelial cells with organoids in SF matrices is capable of inducing spontaneous lumen formation and the development of perfusable microvessels. Such resulting vascular networks are crucial to maintaining organoid viability and enhancing their complex tissue remodeling and differentiation over extended culture times, commonly in excess of 30 days [126]. For example, an overview of intestinal organoid’s applications is given in Figure 10. The degradation kinetic of the SF under controlled conditions also provides a stable structural scaffold for the hydrogel during such prolonged maturation times, enabling complex cellular organization for mature organoids. Such ability to incorporate functioning vasculature is instrumental towards the development of more complex organoid models that closely mimic in vivo tissue structure, e.g., cardiac organoids utilized in regenerative medicine research for myocardial infarction repair studies [13,123,127].

Besides their use in structural growth and development, SF hydrogels offer a robust and healthy platform for the development of complex disease models and drug screening. Organoids cultured in these hydrogels, including those derived from patient colorectal tumors or liver tissue, closely replicate the structure and phenotype of native tissues and in vivo tumors. This high fidelity supports accurate disease modeling across various conditions, such as cancers, neurodegenerative disorders, and inflammatory disorders [17,126]. The stability and biocompatibility of SF help organoids maintain genetic and phenotypic integrity, enabling reliable research on disease mechanisms and therapeutic responses [10]. In drug screening, SF-based systems support high-throughput analysis and real-time monitoring of cellular responses, drug absorption, and toxicity, which are essential for evaluating potential therapies [126].

SF hydrogels have also been used for controlled and sustained release of therapeutics, such as vancomycin in diabetic wound healing, highlighting their value in preclinical drug discovery and personalized medicine [128,129]. Additionally, SF can be processed into various forms, including particles, films, fibers, hydrogels, and 3D scaffolds, expanding its applications in biomedical research [10].

These studies show that SF hydrogels provide a scalable, tunable, and reproducible alternative to Matrigel for organoid culture. SF permits modular incorporation of defined ECM peptides and stiffness/viscoelastic gradients, supports vascularization and maturation of organoids when co-cultured with growth factors or endothelial cells, and allows long-term, stable cultures that are conducive to disease modeling and drug testing. Significantly, SF’s suitability for microfabrication, microfluidics, and bioprinting processes makes it a potential candidate for the translation of organoid platforms into preclinical and industrial testing pathways under regulated environments.

## 5. Challenges and Future Directions

### 5.1. Biological Challenges

Bio-integration of living cells into SF hydrogels is still a major challenge. Crosslinked or dense SF matrices that are highly dense may limit oxygen and nutrient delivery, allowing hypoxic niches that compromise long-term cell viability and function [17]. This has been mostly the case in organoid models, where SF scaffolds increased architectural stability but were still not able to mature completely without additional vascularization or perfusion systems. Immunological considerations introduce additional complexity to biological applications. Although SF is generally biocompatible, but traces of sericin and impurities can provoke immune response, thus its immunogenic properties depend on processing methods. To minimize immunogenicity, diverse processing methods are generally employed. Degumming is one method, where sericin proteins are eliminated by gentle alkaline or enzymatic processes. Aqueous dissolution and dialysis are another procedure. Low-molecular-weight impurities are eliminated with this. Regeneration follows, with controlled drying, lyophilization, or solvent casting being used to maintain structural purity. Refinement procedures involve ultrafiltration, ethanol annealing, or methanol treatment. These steps also reduce residual antigenic moieties more and stabilize the β-sheet crystalline structure. As a result, biocompatibility is increased and risk of inflammation minimized [130,131]. Hydrogels formed by self-assembly of SF typically induce minimal and transient inflammatory responses, similar to those observed with PEG-based matrices, whereas high sericin content can provoke stronger immune stimulation [66,91]. Notably, intentional use of SF–sericin composites has been shown to modulate macrophage polarization during wound healing, indicating that SF’s immunogenicity can be tailored for specific therapeutic objectives [132,133].

### 5.2. Material & Engineering Challenges

Scalability and reproducibility present significant challenges in materials science. Inconsistencies between batches of SF hydrogels often result from differences in silk sources, degumming processes, dissolution methods, and crosslinking techniques, making it difficult to achieve consistent results and scale up production [134,135]. Producing medical-grade SF is also expensive, mainly due to the high costs of purification and sterilization required for clinical use. Adjusting the mechanical properties of hydrogels is complex because they need to match a wide range of tissue stiffness, from soft neural tissue to rigid cartilage, while still maintaining important features like self-healing and biodegradability [136,137,138]. Incorporating engineered living systems, such as probiotics or immune cells, adds further complexity. Ensuring biosafety in these cases requires reliable genetic controls to prevent microorganism escape or uncontrolled cytokine release [139,140,141].

### 5.3. Translational & Regulatory Challenges

Translational SF-derived living hydrogels face several production and regulatory challenges. Large-scale manufacturing must follow good manufacturing practice (GMP) and ensure consistent degradation rates, mechanical properties, and sterility, but these standards are still developing for SF materials [142,143,144]. Regulatory requirements are also demanding. While pure SF hydrogels might be regulated as medical devices, adding living components like stem cells or engineered microbes means they are classified as Advanced Therapy Medicinal Products (ATMPs) or combination biologics, which require stricter approval processes [145,146]. This hybrid classification can significantly delay clinical translation. Additionally, high manufacturing costs and variability in clinical-grade SF processing make it difficult to expand beyond niche commercial markets.

### 5.4. Future Outlook & Opportunities

Despite current limitations, the outlook for SF-based smart living hydrogels remains highly promising. Advances in SF scaffold-driven synthetic biology are enabling new therapeutic platforms, where encapsulated engineered microbes or immune cells act as in situ bio-factories, releasing therapeutic proteins, angiogenic factors, or immunomodulators in a controlled, biomimetic way [84,85,86]. Recent progress in 3D bioprinting and microfluidic engineering is also addressing vascularization challenges by enabling the creation of perfusable SF hydrogel constructs that support organoid maturation and functional tissue regeneration [13,147,148,149]. Meanwhile, hybrid SF composites with conductive nanomaterials such as MXenes or graphene were discovered as novel bioelectronic interfaces for real-time sensing and electrical stimulation of neural and cardiac regeneration [150]. Finally, personalized regenerative medicine has become a reality: SF hydrogel-encapsulated patient-derived iPSCs are being designed to mimic native tissue environments in the attempt to offer personalized implants for cartilage, neural, or bone regeneration [66,151]. Together, these directions place SF as a transformative platform at the intersection of biomaterials, synthetic biology, and regenerative medicine.

After overcoming such challenges through rigorous engineering, optimized protocols, and smart design, SF-derived living hydrogels can become more than a highly technical scholarly niche. These platforms have the potential to become important in clinical settings and could be produced on a large scale. Over the next decade, these advances could lead to personalized regenerative implants and new organoid-based medicines.

## 6. Conclusions

SF-derived smart living hydrogels are a new type of bioactive and adaptable scaffold with clear clinical potential. These materials bring together advances from both regenerative medicine and organoid engineering. In regenerative medicine, they provide structural support, send dynamic signals, and help with cell-based therapies. For organoid engineering, they mimic the properties of the ECM to support organoid growth, blood vessel formation, and functional development. Compared to traditional hydrogels, SF-based scaffolds offer better mechanical strength, a structure that can be absorbed by the body, and the ability to be chemically customized. They can also hold and protect complex living systems, such as stem cells, organoid precursors, immune cells, and engineered microbes. These hydrogels can change their structure in response to different triggers like pH, enzymes, temperature, or light. This lets them adapt in the body, release helpful signals when needed, and develop alongside healing tissues or growing organoids. Their natural origin, compatibility with the body, and history of Food and Drug Administration (FDA)-approved uses, such as silk sutures and surgical meshes, give SF-derived hydrogels a regulatory advantage and make them more likely to be used in clinical settings than many synthetic materials.

A key advancement of SF-based smart living hydrogels is their function as a bidirectional translational bridge. These hydrogels help move organoid models from the lab to clinical-grade therapeutic implants. They also support the shift from clinical regenerative scaffolds to advanced organoid platforms that better mimic real disease conditions for research and drug discovery. By combining SF engineering, synthetic biology, and organoid science, regenerative medicine may soon use SF-based implants that both repair tissue and interact with the immune system, offering tailored therapeutic signals for each patient. Additionally, SF-based organoid scaffolds can be developed into reproducible and standardized precision medicine platforms by integrating patient-derived cells with engineered matrices for disease-specific modeling. Collectively, these programmable and responsive systems represent a shift in biomaterials, moving from passive supports to intelligent, translational scaffolds that connect basic research with clinical applications.

## Figures and Tables

**Table 1:** The design toolkit.

Design Axis	Capabilities	Examples	Ref.
Matrix Engineering	Mechanical tuning, biodegradability, biochemical functionalization	SF–gelatin bioinks, PASCH for wound healing, SF/BMP-2/VEGF for bone regeneration	[39,41,49]
Living Integration	Encapsulation of MSCs, organoid progenitors, therapeutic cells/microbes	SF hydrogels for stem cell encapsulation and recently emerging concepts of embedded therapeutic cells	[50,51]
Smart Responsiveness	Stimuli-triggered changes, self-healing, dynamic degradation and release	pH/glucose responsive systems, enzyme-crosslinked conformational hydrogels, CO_2_ gelation methods	[52,53,56,57,58]

## Data Availability

No new data were created or analyzed in this study. Data sharing is not applicable to this article.
